# Supplementary material for: Entropy scaling for diffusion coefficients in fluid mixtures
Source: Nat Commun. 2025 Mar 17;16:2611. doi: 10.1038/s41467-025-57780-z (PMC11914492; doi:10.1038/s41467-025-57780-z)
Supplement: Supplementary file 1 — Supplementary Information [file 41467_2025_57780_MOESM1_ESM.pdf]

Supplementary Information

Entropy scaling for diffusion coefficients in fluid mixtures

Sebastian Schmitt, Hans Hasse, and Simon Stephan

Laboratory of Engineering Thermodynamics (LTD),
RPTU Kaiserslautern, Kaiserslautern, Germany

simon.stephan@rptu.de

Suppl. Note 1 Scaled Chapman-Enskog diffusion coefficients

Suppl. Note 1.1 Infinite-dilution diffusion coefficient

The scaled Chapman-Enskog infinite-dilution diffusion coefficient $D^{\infty,\circ}_{\mathrm{CE},i}$ is only a function of the temperature and given as

$$D^{\infty,\circ}_{\mathrm{CE},i} = \frac{3}{8\sqrt{\pi}} \frac{1}{\sigma_{ij}^2 \Omega^{(1,1)}} \left(T \left(\frac{\mathrm{d}B}{\mathrm{d}T} \right) + B \right)^{2/3}, \quad (1)$$

where B is the second virial coefficient of the solvent at a given temperature (which is computed from the EOS model), σ_{ij} is the cross-interaction Lennard-Jones size parameter, and $\Omega^{(1,1)}_{ij} = \Omega^{(1,1)}(T \mathrm{k_B} \varepsilon_{ij}^{-1})$ is the collision integral for diffusion [1, 2]. The Lennard-Jones parameters σ_{ij} and ε_{ij} are calculated according to the Lorentz-Berthelot combining rules [3, 4] from the pure component interaction parameters as

$$\sigma_{ij} = \frac{\sigma_i + \sigma_j}{2} \quad \text{and} \quad (2)$$

$$\varepsilon_{ij} = \xi_{ij} \sqrt{\varepsilon_j \varepsilon_i}, \quad (3)$$

where ξ_{ij} is a state-independent mixture parameter to establishing a non-ideality in the system The Lennard-Jones parameters σ_i, σ_j, ε_i, and ε_j of a given pure (possibly real) component are calculated by the corresponding states principle with the Lennard-Jones fluid as reference from the critical temperature $T_{\mathrm{c},j}$ and the critical pressure $p_{\mathrm{c},j}$ of the solvent as $\varepsilon_j = T_{\mathrm{c},j} \mathrm{k_B}/1.321$ and $\sigma_j = (\varepsilon_j 0.129/p_{\mathrm{c},j})^{1/3}$.

Suppl. Note 1.2 Diffusion coefficients in mixtures

The mutual diffusion coefficient in gases does not depend on the composition for mixtures and the Fickian and the Maxwell-Stefan diffusion coefficients are equal, i.e. $D_{ij} = Đ_{ij}$. The Chapman-Enskog self-diffusion coefficients in the mixture $D^{\circ}_{i,\mathrm{CE}}(\underline{x})$ and $D^{\circ}_{j,\mathrm{CE}}(\underline{x})$ are calculated according to Miller and Carman [5] as

$$\frac{1}{D^{\circ}_{\mathrm{CE},i}} = \frac{x_i}{D^{\mathrm{pure},\circ}_{\mathrm{CE},i}} + \frac{x_j}{D^{\infty,\circ}_{\mathrm{CE},i}} \quad \text{and} \quad (4)$$

$$\frac{1}{D^{\circ}_{\mathrm{CE},j}} = \frac{x_j}{D^{\mathrm{pure},\circ}_{\mathrm{CE},j}} + \frac{x_i}{D^{\infty,\circ}_{\mathrm{CE},j}}, \quad (5)$$

where the Rosenfeld-scaled self-diffusion coefficients of the pure components $D^{\mathrm{pure},\circ}_{\mathrm{CE},i}$ and $D^{\mathrm{pure},\circ}_{\mathrm{CE},j}$ are adapted from Ref. [6] as

$$D^{\mathrm{pure},\circ}_{\mathrm{CE},i} = \frac{3}{8\sqrt{\pi}} \frac{1}{\sigma_i^2 \Omega^{(1,1)}_i} \left(T \left(\frac{\mathrm{d}B}{\mathrm{d}T} \right) + B \right)^{2/3}, \quad (6)$$

and the Rosenfeld-scaled infinite-dilution diffusion coefficients $D^{\infty,\circ}_{\mathrm{CE},i}$ and $D^{\infty,\circ}_{\mathrm{CE},j}$ are given by Eq. (1). For the Maxwell-Stefan diffusion coefficient in the mixture, the corresponding Chapman-Enskog diffusion coefficient is given as

$$Đ^{\circ}_{\mathrm{CE},ij} = \frac{3}{8\sqrt{\pi}} \frac{1}{\sigma_{ij}^2 \Omega_{ij}^{(1,1)}} \left(T \left(\frac{\mathrm{d}B}{\mathrm{d}T} \right) + B \right)^{2/3}, \tag{7}$$

where B is the second virial coefficient of the mixture calculated as

$$B = x_i^2 B_i + x_i x_j B_{ij} + x_j^2 B_j, \tag{8}$$

where B_i and B_j are the second virial coefficients of the pure components and B_{ij} is cross second virial coefficient.

For determining the minimum of $\Lambda^{\circ}_{\mathrm{CE}}$, i.e. $\min(\Lambda^{\circ}_{\mathrm{CE}})$, the mixing rule proposed by Miller and Carman [5] is employed, for both the self-diffusion coefficients as well as for the Maxwell-Stefan diffusion coefficient as

$$\frac{1}{\min D^{\circ}_{\mathrm{CE},i}} = \frac{x_i}{\min D^{\mathrm{pure},\circ}_{\mathrm{CE},i}} + \frac{x_j}{\min D^{\infty,\circ}_{\mathrm{CE},i}}, \tag{9}$$

$$\frac{1}{\min D^{\circ}_{\mathrm{CE},j}} = \frac{x_i}{\min D^{\infty,\circ}_{\mathrm{CE},j}} + \frac{x_j}{\min D^{\mathrm{pure},\circ}_{\mathrm{CE},j}}, \tag{10}$$

$$\frac{1}{\min Đ^{\circ}_{\mathrm{CE},ij}} = \frac{x_i}{\min D^{\infty,\circ}_{\mathrm{CE},j}} + \frac{x_j}{\min D^{\infty,\circ}_{\mathrm{CE},i}}. \tag{11}$$

Suppl. Note 2 Simulation details

Molecular dynamics (MD) simulations were carried out in this work for both model systems as well as real substance systems. In both cases, the infinite-dilution diffusion coefficients were sampled. All simulations were carried out with the simulation engine ms2. The Lennard-Jones potential between two particles i and j is defined as

$$u_{ij} = 4\varepsilon_{ij} \left[\left(\frac{\sigma_{ij}}{r_{ij}} \right)^{12} - \left(\frac{\sigma_{ij}}{r_{ij}} \right)^{6} \right], \tag{12}$$

where u_{ij} is their potential energy, r_{ij} the distance between both particles, and σ_{ij} and ε_{ij} the size and energy parameters of the particles, respectively. For all three systems, the size parameters of both components were equal, i.e. $\sigma_2 = \sigma_1$. The energy parameter was $\varepsilon_2 = 0.9\varepsilon_1$ for the first system and $\varepsilon_2 = 0.5\varepsilon_1$ for the two other systems. The potential parameters for the interaction of unlike particles were calculated according to Eqs. (2) and (3) with $\xi_{12} = 1.2$ for the first system, $\xi_{12} = 1$ for the second system, and $\xi_{12} = 0.85$ for the third system. All three systems show a strongly non-ideal behavior, a high-boiling azeotrope (first mixture), a supercritical low-boiling component (second mixture), and a miscibility gap (third mixture).

For each system, simulations at 114 state points in the gas, liquid, metastable vapor-liquid, metastable solid-liquid, and supercritical region (of the solvent) were

carried out (see Fig. 2 in the main text). For each temperature-pressure pair, three simulations in the vicinity of the infinite dilution limit ($x_2 = 0.001, 0.005, 0.01\,\mathrm{mol\,mol^{-1}}$) were carried out. The three simulations were used to extrapolate to infinite dilution ($x_2 \to \infty$) in a post-processing (see below).

The simulations were performed with the software *ms*2[7]. Each simulation consisted of 5,000 particles. The Gear-predictor-corrector algorithm was used for time integration with a time step of $\Delta\tau = 0.001\,\sigma\sqrt{\varepsilon^{-1}M}$. The simulations were conducted in the isochoric-isothermal (NVT) ensemble with 10^5 equilibration time steps and $5 \cdot 10^6$ production time steps. Periodic boundary conditions were applied in all directions. The self-diffusion coefficient of component 2 D_2 was sampled using the Green-Kubo formalism with a correlation length of 10^4 time steps for $\rho \geq 0.1\,\sigma_1^{-3}$ and 10^5 time steps for $\rho < 0.1\,\sigma_1^{-3}$. The infinite-dilution diffusion coefficient D_2^∞ at a given temperature-density pair was computed by linear extrapolation from the results at finite dilution, cf. Fig. 1. The configurational entropy s_conf was determined in the

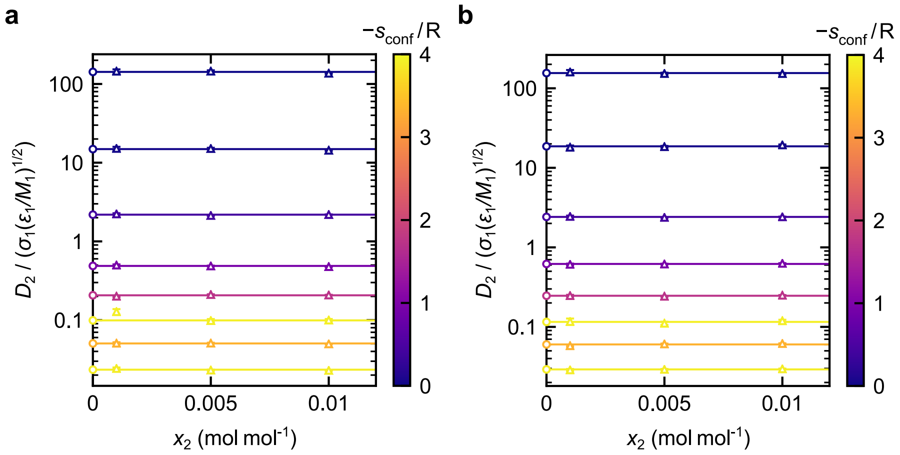

Suppl. Fig. 1 Extrapolation to infinite dilution. Self-diffusion coefficient D_2 as function of the mole fraction x_2 for eight state points (exemplarily chosen) in two Lennard-Jones systems with a) $\sigma_2 = \sigma_1$, $\varepsilon_2 = 0.9\varepsilon_1$, $\varepsilon_{12} = 1.2\sqrt{\varepsilon_1\varepsilon_2}$ (a) and $\sigma_2 = \sigma_1$, $\varepsilon_2 = 0.5\varepsilon_1$, and $\varepsilon_{12} = 0.85\sqrt{\varepsilon_1\varepsilon_2}$ (b). Triangles are the simulation results for the self-diffusion coefficient D_2, the circles are the extrapolated infinite-dilution diffusion coefficients D_2^∞, and the lines represent the linear extrapolations. The color indicates the configurational entropy. Source data are provided as a Source Data file.

simulations using the relation

$$s_\mathrm{conf} = \frac{u_\mathrm{conf}}{T} - \frac{p}{\rho T} - \sum_{i=1}^{2} \frac{\mu_{\mathrm{conf},i}}{T}, \tag{13}$$

where u_conf is the configurational internal energy and $\mu_{\mathrm{conf},i}$ is the chemical potential of component i. The chemical potentials were sampled using Widom's test particle method [8]. The results for the system with $\varepsilon_2 = 0.9\varepsilon_1$ and $\xi_{12} = 0.85$ are presented

in the main body of this work. The numeric values for all three studied systems are provided in the electronic Supporting Information.

For the real substance systems, three mixtures were studied, namely acetone + isobutane, ethanol + chlorine, and benzene + isobutane – using the same methodology. The component-specific force fields from Refs. [9–13] – taken from the MolMod database [14] – were used. In total, 29 state points were investigated in the liquid, gas, and supercritical regions for each real substance binary system. The time step was 0.329 fs for simulations with $\rho > 8.9\,\mathrm{mol\,L^{-1}}$ and 0.987 fs elsewise. Each simulation consisted of 4096 molecules – 4 molecules of the highly diluted component and 4092 molecules of the solvent ($x_1 \approx 0.001\,\mathrm{mol\,mol^{-1}}$). The simulations were equilibrated for $5 \cdot 10^5$ time steps and the actual production run consisted of $6 \cdot 10^6$ time steps. The self-diffusion coefficient of the solvent D_1 was sampled using the Green-Kubo formalism with a correlation length 10^4 time steps. The chemical potential was sampled using Widom's test particle method to calculate the configurational entropy (see Eq. (13)). The second virial coefficient as well as its temperature derivative was sampled for all considered temperatures.

Suppl. Note 3 Component-specific EOS models

The entropy scaling framework proposed in this work for the prediction of mixture diffusion coefficients can be coupled with practically any molecular-based equation of state (and with minor adjustments also with empirical multiparameter EOS [15], e.g. regarding the molecular property parameters m, ε, σ). In this work, we used the Kolafa-Nezbeda EOS for modeling the Lennard-Jones model mixtures and the PC-SAFT EOS for modeling the real substance mixtures. The Kolafa-Nezbeda EOS was found to be the most accurate and robust EOS for modeling thermodynamic properties of the Lennard-Jones fluid [16, 17]. The Kolafa-Nezbeda EOS, originally published for the pure component LJ fluid, was extended in our implementation to mixtures using van der Waals one fluid mixing rule and the modified Lorentz-Berthelot combination rules (cf. Eqs. 2 and 3). Using the latter, the binary interaction parameter xsi was directly adopted in the EOS model, which makes the theory fully predictive in this case. Also the PC-SAFT EOS is known to often yield good predictions for thermodynamic mixture properties [18, 19].

The component-specific PC-SAFT EOS models for the real substances were taken from Refs. 20–24. The parameters are given in Table 1. The binary interaction parameters ξ_{12} of all real substance systems was unity for the systems n-hexane + n-dodecane, toluene + n-hexane, and 2-propanol + n-heptane; for acetone + chloroform, it was adjusted to $\xi_{12} = 1.02$; for nitrobenzene + n-hexane, it was adjusted to $\xi_{12} = 1.05$; for acetone + acetonitrile, it was adjusted to $\xi_{12} = 0.97$. For the parametrization, only phase equilibrium data was used, i.e. no transport property data. Hence, the mixture diffusion coefficients are described in a predictive way. For applications, the binary interaction parameter could alos be adjusted to experimental diffusion coefficient data to improve the model performance.

Suppl. Table 1 Component-specific PC-SAFT EOS parameters from the literature used in the present work. The columns indicate (from left to right): The substance name, segment diameter σ, segment dispersion energy ε, chain length parameter m, dipole moment D, association volume κ_{AB}, and association strength ε_{AB}.

substance	σ	ε/k_B	m	μ	κ_{AB}	ε_{AB}/k_B	Ref
	Å	K		D		K	
n-hexane	3.7983	236.77	3.0576				20
n-heptane	3.8049	238.40	3.4831				20
n-dodecane	3.8959	249.21	5.306				20
acetone	3.2557	253.406	2.77409				21
acetonitrile	3.3587	313.04	2.2661				22
chloroform	3.4709	271.63	2.5038				21
nitrobenzene	3.6415	344.88	3.1442				23
toluene	3.7169	285.69	2.8149				20
2-propanol	3.38	212.32	2.685	1.7	0.024675	2253.9	24

Suppl. Note 4 Entropy scaling models

The entropy scaling limiting case model parameters (pure component and pseudo-pure component) used in this work are reported in Table 2. Additionally, the references of the experimental data used for the parameter adjustment are given. In all cases, two component-specific parameters were used due to small number of experimental data available (in two cases, one parameter was used). In cases where more reference data are available, functions with more parameters can be used, cf. Ref. [6].

Suppl. Table 2 Component-specific entropy scaling parameters used in the present work. The columns indicate (from left to right): the system, the property, the parameters $\alpha_{2,i}$ and $\alpha_{3,i}$ as well as the reference where experimental data were taken from.

system	property	$\alpha_{2,i}$	$\alpha_{3,i}$	Ref. data
n-hexane (1) + n-dodecane (2)	D_1	-2.5414	-1.9186	25
	D_2	-4.3257	-3.2885	
	D_1^∞	0.0	-5.3751	26
	D_2^∞	0.0	-3.1396	
acetone (1) + chloroform (2)	D_1	-0.8935	-1.9973	25
	D_2	-1.1496	-2.0841	
	D_1^∞	-3.3309	-1.7752	27
	D_2^∞	-1.9979	-1.6190	
nitrobenzene (1) + n-hexane (2)	D_1	21.2108	-9.9493	25
	D_1^∞	12.7201	-9.3538	28
toluene (1) + n-hexane (2)	D_1^∞	-4.0978	-1.0285	29
	D_2^∞	-2.6050	-1.5678	
toluene (1) + acetonitrile (2)	D_1^∞	-0.1904	-1.5008	30
	D_2^∞	-2.8591	-1.7467	
2-propanol (1) + n-heptane (2)	D_1^∞	-11.9703	0.2606	31
	D_2^∞	5.4598	-2.9687	

Suppl. Note 5 Comparison of the scaling behavior of pure component self-diffusion coefficients and infinite-dilution diffusion coefficients

For the Lennard-Jones model systems, a quasi-universality is observed using the applied scaling, which is evident by the representation of $\widehat{D}_2^{\infty,\circ}$ by the global model from Ref. 6, which was only fitted to pure-component self-diffusion coefficient data. Hence, the correlation developed in Ref. 6 using pure-component self-diffusion coefficient data of the Lennard-Jones fluid also describes the infinite-dilution diffusion coefficient data from this work.

In Fig. 2, the scaling of the pure component self-diffusion coefficient is compared with the scaling of the infinite-dilution diffusion coefficient – for two of the studied LJ systems. Both CE-scaled diffusion coefficients show a monovariate behavior over the entire range of configurational entropies and all temperatures. To quantify this, a measure Δ_Λ was defined as

$$\Delta_\Lambda = \sqrt{\frac{1}{N_\Lambda}\sum_i^{\mathrm{N}}(\Lambda_{\mathrm{sim},i} - \Lambda_{\mathrm{mod},i})^2}, \tag{14}$$

where $\Lambda \in \{\widehat{D}_2^{\mathrm{pure},\circ}, \widehat{D}_2^{\infty,\circ}\}$, N_Λ is the respective number of simulation data points, $\Lambda_{\mathrm{sim},i}$ are diffusion coefficients obtained from the simulations, and $\Lambda_{\mathrm{mod},i}$ the values calculated from the entropy scaling model. The obtained values are $\Delta_{\widehat{D}_2^{\mathrm{pure},\circ}} \approxeq 1.244$ and $\Delta_{\widehat{D}_2^{\infty,\circ}} \approxeq 1.254$ for first system and $\Delta_{\widehat{D}_2^{\mathrm{pure},\circ}} \approxeq 1.244$ and $\Delta_{\widehat{D}_2^{\infty,\circ}} \approxeq 1.254$ for the second Lennard-Jones system. Hence, the infinite-dilution diffusion coefficient data yields a monovariate behavior – with the same extent as the pure component self-diffusion coefficient. This support the perspective that the infinite dilution state can be considered as a pseudo-pure component.

Suppl. Note 6 Scaling behavior of the infinite-dilution diffusion coefficient

Fig. 3 shows the simulation results for two additional Lennard-Jones mixture. The results confirm the findings of the main part: in both mixtures, the scaled infinite-dilution diffusion coefficient shows a monovariate function with respect to the scaled configurational entropy.

Fig. 4 shows the simulation results for the real substance system benzene + isobutane. The simulation state points cover the gas, liquid, and the supercritical region. The scaled infinite-dilution diffusion coefficient of benzene $\widehat{D}_1^{\infty,\circ}$ shows a monovariate behavior with respect to the reduced configurational entropy $\tilde{s}_{\mathrm{conf}}$. The results confirm the validity of the entropy scaling methodology introduced in this work for real substance systems.

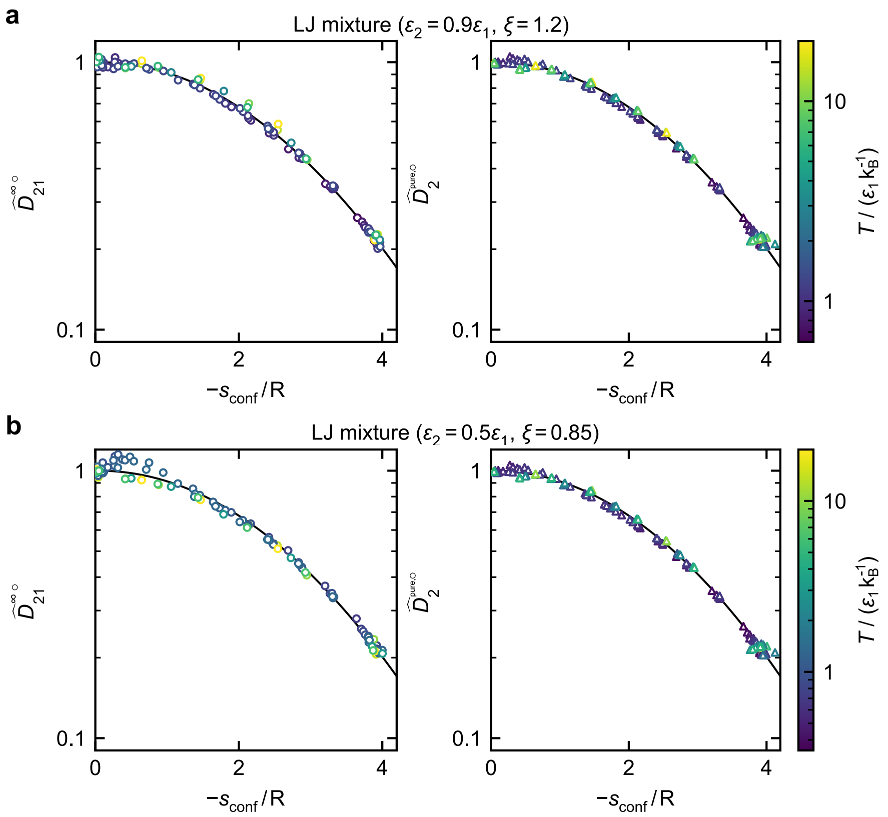

Suppl. Fig. 2 Entropy scaling of infinite-dilution diffusion coefficients. Scaled infinite-dilution diffusion coefficient $\widehat{D}_2^{\infty,\circ}$ (left) and self-diffusion coefficient $\widehat{D}_2^{\mathrm{pure},\circ}$ (right) for two Lennard-Jones systems (a and b) as a function of the configurational entropy s_{conf}. The symbols are simulation results from this work ($\widehat{D}_2^{\infty,\circ}$) and from Ref. 6 ($\widehat{D}_2^{\mathrm{pure},\circ}$). The color of the symbols indicates the temperature. The black solid line is the entropy scaling model. Source data are provided as a Source Data file.

Experimental infinite dilution self-diffusion coefficient data in a large range of states (as considered for the model systems) are unfortunately not available. For the system toluene + n-hexane, experimental data of infinite-dilution diffusion coefficients at different pressures and temperatures are available, but only for the liquid phase. Fig. 5 shows the scaled infinite-dilution diffusion coefficients of the system toluene (1) + n-hexane (2) as function of the reduced configurational entropy, i.e. $\widehat{D}_1^{\infty,\circ}(\tilde{s}_{\mathrm{conf}})$ and $\widehat{D}_2^{\infty,\circ}(\tilde{s}_{\mathrm{conf}})$. Additionally, the entropy scaling models (lines) are shown for both cases. For each case, two system-specific parameters were adjusted (see Table 2). For the system toluene + n-hexane, experimental data for both infinite-dilution diffusion coefficients are available at different temperatures and pressures (which is very seldom). Thus, this system is well suited to demonstrate the scaling of the infinite-dilution diffusion coefficients. All scaled diffusion coefficients lie on a single curve, i.e. show a monovariate behavior with respect to $\tilde{s}_{\mathrm{conf}}$.

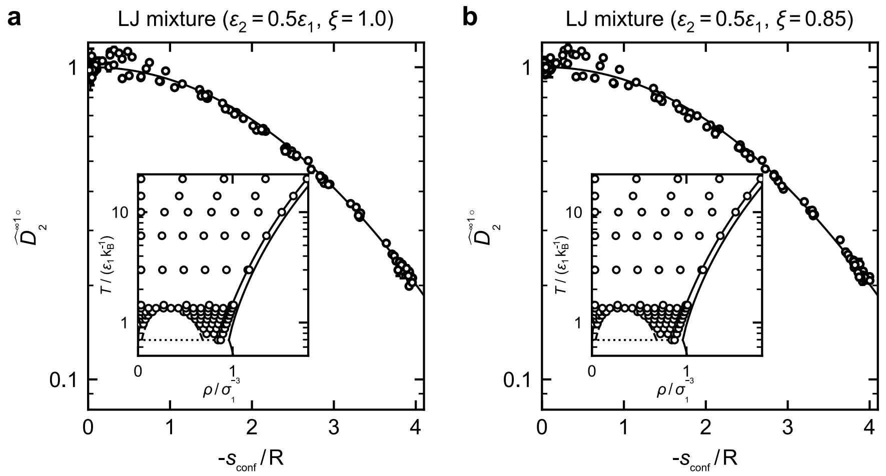

Suppl. Fig. 3 Entropy scaling of infinite-dilution diffusion coefficients in two Lennard-Jones mixtures. a) $\varepsilon_2 = 0.5\,\varepsilon_1$, $\varepsilon_{12} = 1\,\xi_{12}$; b) $\varepsilon_2 = 0.5\,\varepsilon_1$, and $\varepsilon_{12} = 0.85\,\xi_{12}$ (both: $\sigma_2 = \sigma_1$). Scaled infinite-dilution diffusion coefficient of component 2 $\widehat{D}_2^{\infty,\circ}$ as a function the reduced configurational entropy s_{conf}/R. The line indicates the entropy scaling model. Symbols are MD simulation data from this work. The inset shows the simulation state points in the temperature-density phase diagram of component 1. Therein, solid lines indicate the phase envelopes from Refs. 32, 33. Source data are provided as a Source Data file.

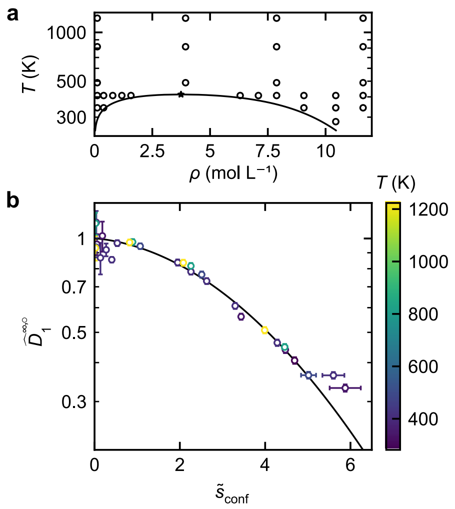

Suppl. Fig. 4 Scaling behavior of the infinite-dilution diffusion coefficient of benzene in the system benzene (1) + isobutane (2). a) Simulation state points (symbols) in the temperature-density phase diagram of the solvent isobutane. The line indicates the vapor-liquid equilibrium and the star the critical point as calculated from the PC-SAFT EOS [20]. b) Scaled diffusion coefficient of benzene infinitely diluted in isobutane $\widehat{D}_1^{\infty,\circ}$ as a function of the configurational entropy $\tilde{s}_{\mathrm{conf}}$. The symbols are the simulation results (color indicates the temperature) and the line the entropy scaling model (fitted to the simulation results). Source data are provided as a Source Data file.

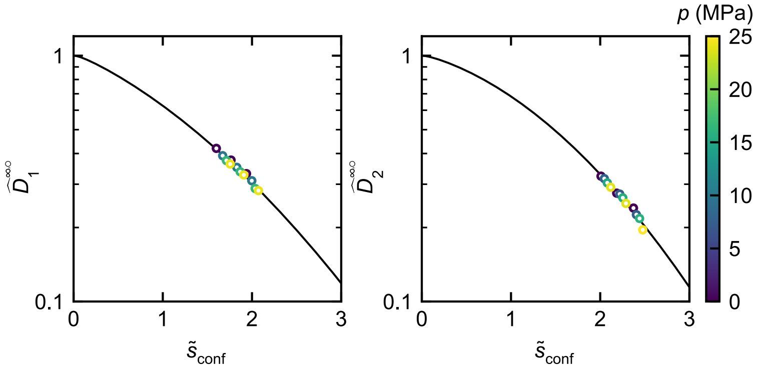

Suppl. Fig. 5 Scaling behavior of the infinite-dilution diffusion coefficients in the system toluene (1) + n-hexane (2) as function of the reduced configurational entropy $\tilde{s}_{\mathrm{conf}}$. Left: Scaled diffusion coefficient of toluene infinitely diluted in n-hexane $\widehat{D}_1^{\infty\circ}$. Right: Scaled diffusion coefficient of n-hexane infinitely diluted in toluene $\widehat{D}_2^{\infty\circ}$. The line indicates the system-specific entropy scaling model (parameters given in Table 2). The color indicates the pressure. Source data are provided as a Source Data file.

Suppl. Note 7 Additional entropy scaling results of the Lennard-Jones mixtures

Fig. 6 shows the application of the entropy scaling model to the Lennard-Jones mixture with $\varepsilon_2 = 0.9\varepsilon_1$ and $\varepsilon_{12} = 1.2\sqrt{\varepsilon_1 \varepsilon_2}$ at different pressures (corresponding to Fig. 4 of the main body). The predictions from the entropy scaling model are compared

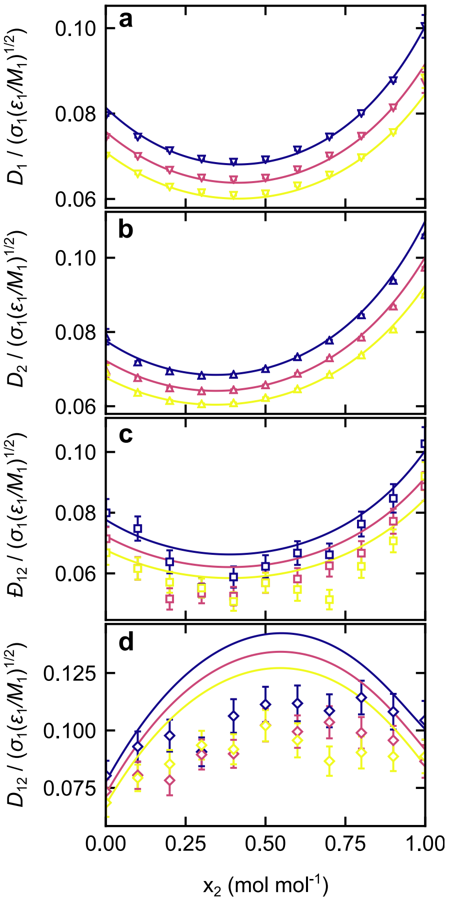

Suppl. Fig. 6 Entropy scaling predictions at different pressures. Diffusion coefficients in the Lennard-Jones system with $\sigma_2 = \sigma_1$, $\varepsilon_2 = 0.9\varepsilon_1$, and $\varepsilon_{12} = 1.2\sqrt{\varepsilon_1 \varepsilon_2}$ as a function of the mole fraction x_2 at $T = 0.92\,\mathrm{k_B}\varepsilon_1^{-1}$. a) Self-diffusion coefficient of component 1 D_1; b) Self-diffusion coefficient of component 2 D_2; c) Maxwell-Stefan diffusion coefficient $Đ_{12}$; d) Fickian diffusion coefficient D_{12}. Lines are the predictions from the entropy scaling model. Symbols are simulation results from Ref. 34. The entropy scaling model was used in combination with the Kolafa-Nezbeda EOS[35]. The colors indicate the pressure $p \in \{0.13, 0.26, 0.39\}\,\sigma_1^3\varepsilon_1^{-1}$ (yellow to dark purple). Source data are provided as a Source Data file.

to simulation data from Ref. 34. The results for different pressures are very similar to those for different temperatures (see main body of this work): The agreement between

the predictions from the entropy scaling model and simulation data is very good for both self-diffusion coefficients. For the mutual diffusion coefficients, some deviations are observed.

Suppl. Note 8 Comparison of entropy scaling to the Vignes and Darken models

The performance of the entropy scaling model is compared to established empirical models. The Vignes equation [36] is an often applied, simple model for calculating Maxwell-Stefan diffusion coefficients in mixtures based on the infinite-dilution diffusion coefficients. It is written as

$$Đ_{ij} = \left(D_i^{\infty}\right)^{x_j} \left(D_j^{\infty}\right)^{x_i} . \tag{15}$$

Besides the Vignes model, the generalized Darken model[37] is often applied and found to be superior in some cases [38]. For binary mixtures, it is defined as

$$Đ_{ij} = x_i \left(x_i^{(\mathrm{m})} D_j^{\infty} + x_j^{(\mathrm{m})} D_j^{(\mathrm{pure})}\right) + x_j \left(x_i^{(\mathrm{m})} D_i^{(\mathrm{pure})} + x_j^{(\mathrm{m})} D_i^{\infty}\right) , \tag{16}$$

where $x_i^{(\mathrm{m})}$ and $x_j^{(\mathrm{m})}$ are the mass fractions of the components i and j, respectively. Both the Vignes and the Darken model only require information on the limiting case diffusion coefficients, like the entropy scaling model proposed in this work.

In Fig. 7, the predictions from the entropy scaling model proposed in this work are compared to the results from the Vignes model and the generalized Darken model for both Lennard-Jones mixtures. Both mixtures show a strongly non-ideal behavior (see main text and above). For both mixtures, the entropy scaling model provides a reasonable description of the Maxwell-Stefan diffusion coefficient in the mixture. The Vignes and Darken models are not able to capture the trend of $Đ_{12}$ in the first Lennard-Jones system (see Fig. 7a). For the second system (see Fig. 7b), the predictions by the Vignes equation show a wrong curvature compared to the simulation data and, most importantly, do not capture the liquid-liquid equilibrium (LLE). Both empirical models (Vignes and Darken) do not comprise information on the liquid-liquid miscibility gap. The entropy scaling model proposed in this work, on the other hand, inherently captures the LLE due to the coupling with the EOS model and also describes the diffusion coefficients of the coexisting phases, metastable phases, supercritical phases etc. However, no computer experiment data is available for the coexisting phase diffusion coefficients for validation.

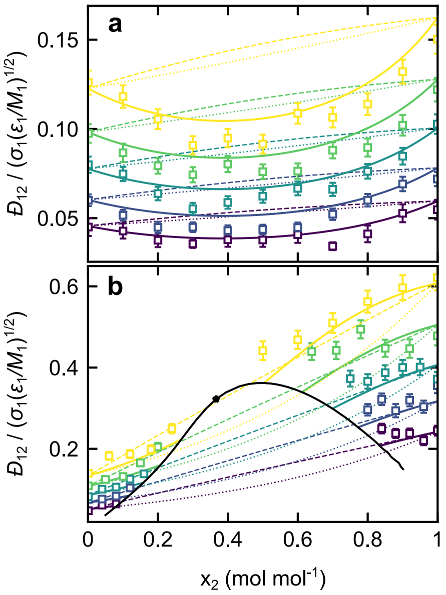

Suppl. Fig. 7 Comparison of MD reference data to the entropy scaling model proposed in this work, the Vignes model, and the Darken model for the Maxwell-Stefan diffusion coefficient. Results for two binary Lennard-Jones systems (a: $\sigma_2 = \sigma_1$, $\varepsilon_2 = 0.9\varepsilon_1$, $\varepsilon_{12} = 1.2\sqrt{\varepsilon_1\varepsilon_2}$, b: $\sigma_2 = \sigma_1$, $\varepsilon_2 = 0.5\varepsilon_1$, $\varepsilon_{12} = 0.85\sqrt{\varepsilon_1\varepsilon_2}$) as a function of the mole fraction x_2 at $p = 0.13\,\sigma_1^3\varepsilon_1^{-1}$ (a) and $p = 0.26\,\sigma_1^3\varepsilon_1^{-1}$ (b). Symbols indicate simulation results from Ref. 34. Solid lines are predictions from the entropy scaling model obtained in combination with the Kolafa-Nezbeda EOS. Dotted lines are results from the Vignes model (cf. Eq. (15)) and dashed lines from the generalized Darken model (cf. Eq. (16)). The colors indicate the temperature $T \in \{0.79, 0.855, 0.92, 0.985, 1.05\}\,\mathrm{k_B}\varepsilon_1^{-1}$ (blue to yellow). The black line indicate the liquid-liquid equilibrium diffusion coefficient and the star the critical point. Error bars represent the simulation uncertainty given in Ref. [34]. Source data are provided as a Source Data file.

Suppl. Note 9 Scaled diffusion coefficients in mixtures

Fig. 8 shows the scaling behavior of diffusion coefficients in mixtures for the two Lennard-Jones systems and a real substance system. For the two considered Lennard-Jones systems, both scaled self-diffusion coefficients and the scaled Maxwell-Stefan diffusion coefficient collapse on one line, which is a special feature of the considered Lennard-Jones mixtures. As a result, a single set of parameters is able to describe all three diffusion coefficients. The scattering of the Maxwell-Stefan diffusion coefficients is larger than that for the self diffusion coefficients, which is due to the scattering of the reference data. For the real substance system n-hexane (1) + n-dodecane (2), results for both scaled self-diffusion coefficients $\widehat{D}_1^\circ$ and $\widehat{D}_2^\circ$ are shown. The corresponding, non-scaled results are shown in Fig. 5a of the main text. The data and models for the pure component self-diffusion coefficient and the pseudo-pure component infinite-dilution diffusion coefficient differ significantly. The self-diffusion coefficients in the mixture ($0 < x_2\,/\,\mathrm{mol\,mol^{-1}} < 1$) lie between both curves. The entropy scaling model connects both lines (here at a given T and p) and is thus able to predict these points.

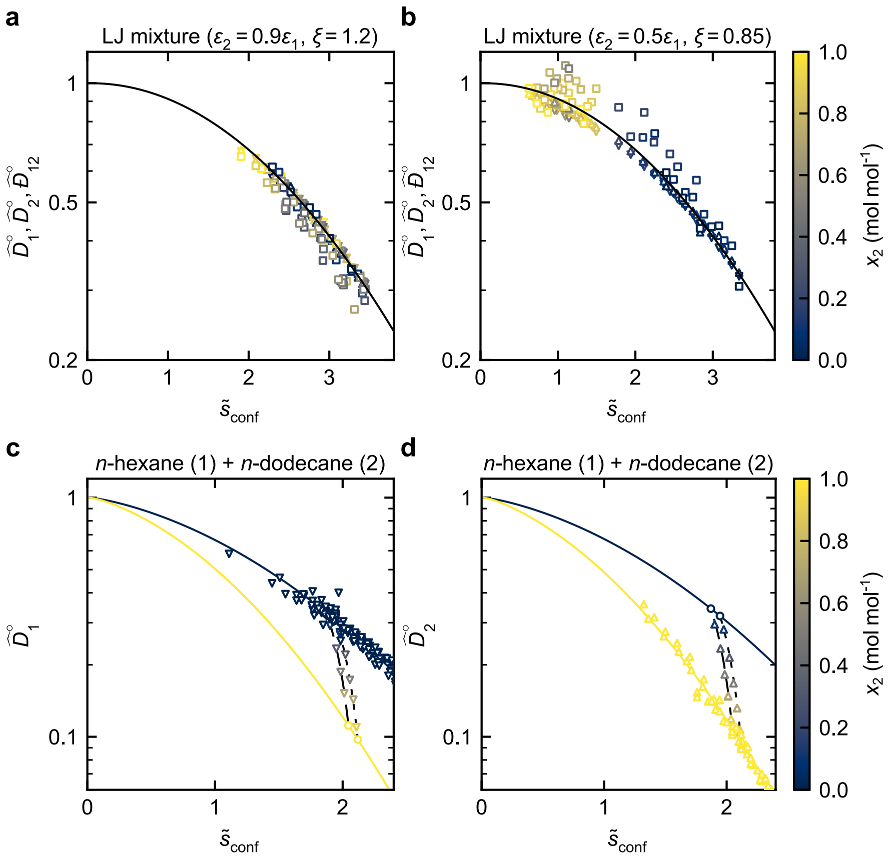

Suppl. Fig. 8 Scaled diffusion coefficients as a function of the configurational entropy $\tilde{s}_{\mathrm{conf}}$ in different mixtures. a and b: Lennard-Jones mixtures. c and d: Mixture n-hexane (1) + n-dodecane (2). Symbols are scaled simulation results (a and b) or experimental data (c and d) [26, 39]. Triangles: Self-diffusion coefficients; squares: Maxwell-Stefan diffusion coefficient; circles: infinite-dilution diffusion coefficients. a + b: The solid line represents the global entropy scaling model for the diffusion coefficients of the Lennard-Jones fluid [6]. c: The yellow and the dark blue lines represent the entropy scaling models for D_1 and D_1^{∞}, respectively. d: The dark blue and the yellow lines represent the entropy scaling model for D_2 and D_2^{∞}, respectively. c and d: Black dotted and dashed lines are results from the entropy scaling model for constant temperature (dotted: $T = 298.15\,K$, dashed: $T = 308.15\,\mathrm{K}$) computed over the entire composition range, i.e. $0 < x_2\,/\,\mathrm{mol\,mol^{-1}} < 1$. Source data are provided as a Source Data file.

The link is primarily established via the entropy of the mixture and the mixing and combination rules built in the entropy scaling model.

Fig. 8 demonstrates the two central elements of the proposed methodology: (1) the infinite-dilution diffusion coefficients, if scaled as proposed in this work, exhibit a monovariate relation and can be treated as a pseudo-pure component, which enables the scaling of that property. The scaling can be used for predicting infinite-dilution diffusion coefficients far beyond the range of available data based on that scaling. (2) The predictions of the diffusion coefficients into the mixture do not follow the

monovariate scaling behavior. Yet, the entropy of the mixture in combination with appropriately designed mixing and combination rules enable the prediction of the diffusion coefficients in the mixture in the scaled variables. For the limiting cases of the pure components and the pseudo-pure components, empirical models for describing the scaled diffusion coefficient are required in this framework. Yet, these models require only very few parameters, e.g. 1 or 2 parameters were used for the real substance cases studied in this work (cf. Table 2). From these few parameters, the model (1) can predict the corresponding diffusion coefficient practically in the entire fluid state region, cf. for example Fig. **2**. For the main part of the novel framework, i.e. the prediction of the different diffusion coefficients in the mixture $D_i(x_j)$, $D_j(x_j)$, $Đ_{ij}(x_j)$, and $D_{ij}(x_j)$ no adjustable parameters are required. The mechanisms for establishing the link between the limiting case diffusion coefficients (pure component and pseudo-pure component) are analogue and consistent to the mechanisms usually used for predicting the viscosity and thermal conductivity of mixtures by entropy scaling. Albeit, significantly more complex in the case of diffusion since different diffusion coefficients are described in a single and consistent framework. However, strong non-idealities of the Maxwell-Stefan diffusion coefficients, especially in binary mixtures of an alcohol and a non-polar substance [40], may not be covered by the proposed framework.

Fig. **9** shows results for a system with an associating component, namely 2-propanol + n-heptane. For associating components, entropy scaling is known to often perform less good [6, 41]. This is also observed here. For the system 2-propanol + n-heptane, significant quantitative deviations between entropy scaling model and the experimental data are observed ($\overline{\delta D_{12}} \approx 50\,\%$). Nevertheless, qualitatively, the minimum of $D_{12}(x_2)$ and the temperature dependency are correctly captured by the model. The entropy scaling shows a prediction with a distinct minimum at $x_2 \approx 0.7\ \mathrm{mol\,mol^{-1}}$ while the experimental data only show slightly non-ideal behavior. The deviations are possibly due to several reasons. The mutual diffusion coefficients of system consisting of an associating and a non-associating component obey a complex behavior at low concentrations of the associating component (here: 2-propanol) [40] which is due to a structuring in the liquid. Additionally, the modeling of other properties in such systems by equations of state also posses challenges. The reasons for the deviations observed in Fig **9** are manifold and require further investigations. The description of these non-idealities, which might be due to local composition phenomena [42], requires modifications of the introduced framework as well as of the underlying EOS models.

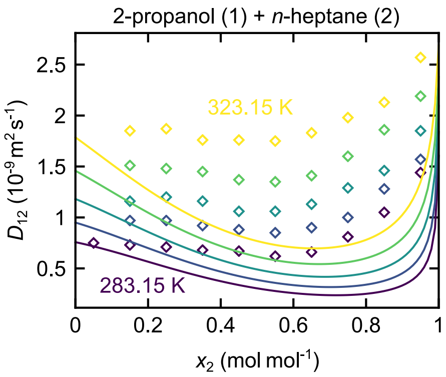

Suppl. Fig. 9 Fickian diffusion coefficients of the mixture 2-propanol (1) + *n*-heptane (2). Predictions by the entropy scaling model as a function of the mole fraction x_2 at $p = 0.1\,\mathrm{MPa}$. Symbols are experimental data from Ref. 43 and lines are model predictions. Source data are provided as a Source Data file.

Suppl. Note 10 Diffusion coefficients at metastable and unstable states

Fig. **10** demonstrates the application of the entropy scaling model to diffusion coefficients at metastable and unstable states in mixtures. Therefore, three lines at constant partial densities of *n*-hexane $\rho_2 = 0.25,\ 0.75,$ or $1.25\,\mathrm{mol\,L^{-1}}$ were calculated by varying the partial density of toluene in the range $0\,\mathrm{mol\,L^{-1}} \leq \rho_1 \leq 5.6\,\mathrm{mol\,L^{-1}}$ at a temperature $T = 560\,\mathrm{K}$, where *n*-hexane is supercritical. This procedure is computationally convenient for calculating metastable and unstable states as the applied PC-SAFT EOS is formulated in the Helmholtz energy a with its fundamental variables T, ρ, and x. All three partial isochores cross the vapor-liquid equilibrium. The corresponding pressures undergo a van der Waals loop with minima and maxima at the spinodals (see Fig. **10**). Evidently, the application of the entropy scaling model in metastable states requires an EOS model that shows a physically reasonable behavior in that region, i.e. a single van der Waals loop, which is the case here. Here, only predictions for the Maxwell-Stefan diffusion coefficient are shown. The Maxwell-Stefan diffusion coefficient smoothly transitions from the liquid phase to the gas phase through the vapor-liquid equilibrium including the metastable and unstable regions. While there is no diffusion coefficient reference data available for validation, these predictions seem physically reasonable.

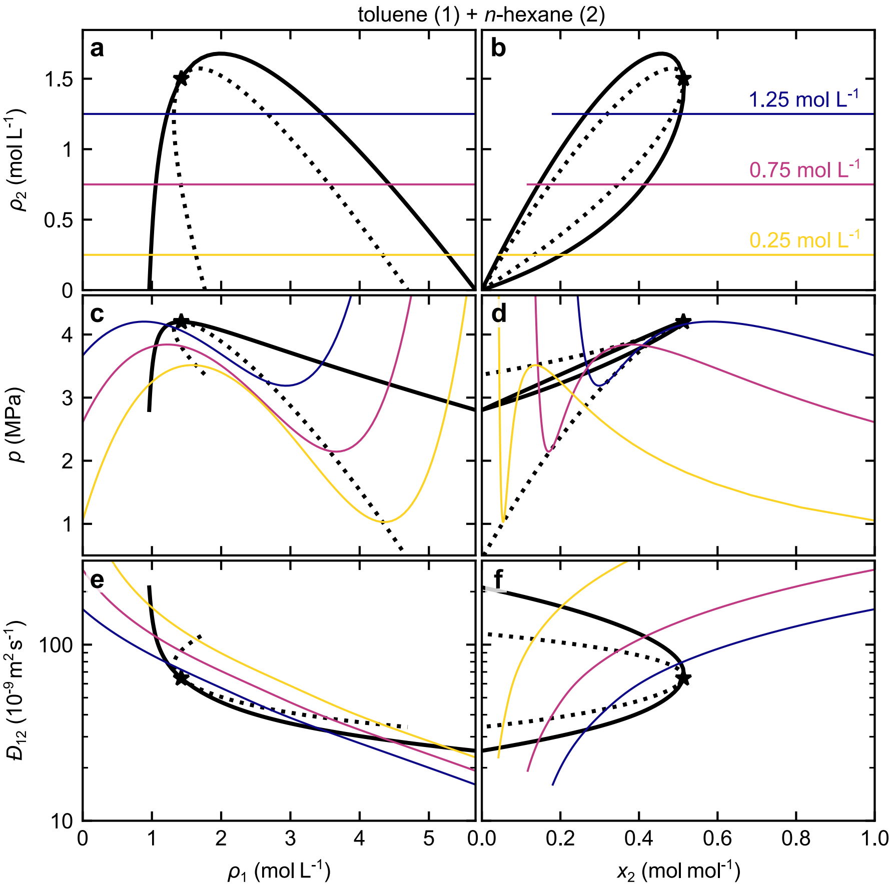

Suppl. Fig. 10 Predictions of the VLE properties and the Maxwell-Stefan diffusion coefficient from the entropy scaling + EOS model in the two-phase vapor-liquid equilibrium region of the binary mixture toluene (1) + *n*-hexane (2). Partial density of n-hexane ρ_2 (top), pressure p (middle), and Maxwell-Stefan diffusion coefficient $Đ_{12}$ (bottom) as a function of the partial density of toluene ρ_1 (a, c, and e) and of the mole fraction x_2 (b, d, and f) at $T = 560\,\mathrm{K}$. The black solid lines correspond to the vapor-liquid binodal, the dotted line to the vapor-liquid spinodal, and the black star indicates the critical point. The three colored lines are lines with constant partial density of n-hexane ρ_2. Source data are provided as a Source Data file.

Supplementary References

[1] Elliott, J. R., Diky, V., Knotts, T. A. & Wilding, W. V. *The properties of gases and liquids* 6th edn (McGraw Hill, New York, Chicago, San Francisco, 2023).

[2] Kim, S. U. & Monroe, C. W. High-accuracy calculations of sixteen collision integrals for Lennard-Jones (12-6) gases and their interpolation to parameterize neon, argon, and krypton. *J. Comput. Phys.* **273**, 358–373 (2014).

[3] Lorentz, H. A. Ueber die Anwendung des Satzes vom Virial in der kinetischen Theorie der Gase. *Ann. Phys.* **248**, 127–136 (1881).

[4] Berthelot, D. Sur le melange des gaz. *C.R. Hebd. Seances Acad. Sci.* **126**, 1703 – 1706 (1898).

[5] Miller, L. & Carman, P. C. Self-diffusion in mixtures. Part 4. – Comparison of theory and experiment for certain gas mixtures. *Trans. Faraday Soc.* **57**, 2143–2150 (1961).

[6] Schmitt, S., Hasse, H. & Stephan, S. Entropy scaling framework for transport properties using molecular-based equations of state. *J. Mol. Liq.* **395**, 123811 (2024).

[7] Fingerhut, R. *et al.* ms2: A molecular simulation tool for thermodynamic properties, release 4.0. *Comput. Phys. Commun.* **262**, 107860 (2021).

[8] Widom, B. Some topics in the theory of fluids. *J. Chem. Phys.* **39**, 2808–2812 (1963).

[9] Guevara-Carrion, G., Janzen, T., Muñoz-Muñoz, Y. M. & Vrabec, J. Mutual diffusion of binary liquid mixtures containing methanol, ethanol, acetone, benzene, cyclohexane, toluene, and carbon tetrachloride. *J. Chem. Phys.* **144**, 124501 (2016).

[10] Eckl, B., Vrabec, J. & Hasse, H. Set of molecular models based on quantum mechanical ab initio calculations and thermodynamic data. *J. Phys. Chem. B* **112**, 12710–12721 (2008).

[11] Stöbener, K., Klein, P., Horsch, M., Küfer, K. & Hasse, H. Parametrization of two-center Lennard-Jones plus point-quadrupole force field models by multicriteria optimization. *Fluid Phase Equilib.* **411**, 33–42 (2016).

[12] Schnabel, T., Vrabec, J. & Hasse, H. Henry's law constants of methane, nitrogen, oxygen and carbon dioxide in ethanol from 273 to 498 K: Prediction from molecular simulation. *Fluid Phase Equilib.* **233**, 134–143 (2005).

[13] Windmann, T., Linnemann, M. & Vrabec, J. Fluid Phase Behavior of Nitrogen + Acetone and Oxygen + Acetone by Molecular Simulation, Experiment and the Peng-Robinson Equation of State. *J. Chem. Eng. Data* **59**, 28–38 (2014).

[14] Stephan, S., Horsch, M. T., Vrabec, J. & Hasse, H. MolMod - an open access database of force fields for molecular simulations of fluids. *Mol. Simul.* **45**, 806–814 (2019).

[15] Span, R. *Multiparameter equations of state* (Springer Berlin Heidelberg, Berlin, Heidelberg, 2000).

[16] Stephan, S., Staubach, J. & Hasse, H. Review and comparison of equations of state for the Lennard-Jones fluid. *Fluid Phase Equilib.* **523**, 112772 (2020).

[17] Antolović, I., Staubach, J., Stephan, S. & Vrabec, J. Phase equilibria of symmetric Lennard-Jones mixtures and a look at the transport properties near the upper critical solution temperature. *Phys. Chem. Chem. Phys.* **25**, 17627–17638 (2023).

[18] Stephan, S., Fleckenstein, F. & Hasse, H. Vapor-liquid interfacial properties of the systems (toluene + CO_2) and (toluene + N_2): Experiments, molecular simulation, and density gradient theory. *J. Chem. Eng. Data* **69**, 590–607 (2024).

[19] Staubach, J., Schwarz, G., Möbius, S., Hasse, H. & Stephan, S. Modeling thermodynamic properties of mixtures of CO_2 + O_2 in the Allam cycle by equations of state. *Int. J. Thermophys.* **44**, 182 (2023).

[20] Gross, J. & Sadowski, G. Perturbed-chain SAFT: An equation of state based on a perturbation theory for chain molecules. *Ind. Eng. Chem. Res.* **40**, 1244–1260 (2001).

[21] Kouskoumvekaki, I. A., von Solms, N., Michelsen, M. L. & Kontogeorgis, G. M. Application of the perturbed chain SAFT equation of state to complex polymer systems using simplified mixing rules. *Fluid Phase Equilib.* **215**, 71–78 (2004).

[22] Spuhl, O., Herzog, S., Gross, J., Smirnova, I. & Arlt, W. Reactive Phase Equilibria in Silica Aerogel Synthesis: Experimental Study and Prediction of the Complex Phase Behavior Using the PC-SAFT Equation of State. *Ind. Eng. Chem. Res.* **43**, 4457–4464 (2004).

[23] Grenner, A., Tsivintzelis, I., Economou, I. G., Panayiotou, C. & Kontogeorgis, G. M. Evaluation of the Nonrandom Hydrogen Bonding (NRHB) Theory and the Simplified Perturbed-Chain−Statistical Associating Fluid Theory (sPC-SAFT). 1. Vapor−Liquid Equilibria. *Ind. Eng. Chem. Res.* **47**, 5636–5650 (2008).

[24] Al-Saifi, N. M., Hamad, E. Z. & Englezos, P. Prediction of vapor-liquid equilibrium in water-alcohol-hydrocarbon systems with the dipolar perturbed-chain SAFT equation of state. *Fluid Phase Equilib.* **271**, 82–93 (2008).

[25] Suárez-Iglesias, O., Medina, I., Sanz, M., Pizarro, C. & Bueno, J. L. Self-diffusion in molecular fluids and noble gases: Available data. *J. Chem. Eng. Data* **60**, 2757–2817 (2015).

[26] Shieh, J. J. C. & Lyons, P. A. Transport properties of liquid n-alkanes. *J. Phys. Chem.* **73**, 3258–3264 (1969).

[27] Anderson, D. K., Hall, J. R. & Babb, A. L. Mutual diffusion in non-ideal binary liquid mixtures. *J. Phys. Chem.* **62**, 404–408 (1958).

[28] D'Agostino, C., Mantle, M. D., Gladden, L. F. & Moggridge, G. D. Prediction of binary diffusion coefficients in non-ideal mixtures from NMR data: Hexane-nitrobenzene near its consolute point. *Chem. Eng. Sci.* **66**, 3898–3906 (2011).

[29] Chen, J. & Zhang, J. Determination of liquid diffusion coefficients for binary systems by improved metallic diaphragm cell method. *J. Chem. Eng. Chin. Univ.* **6**, 196 – 201 (1992).

[30] Awan, M. A. & Dymond, J. H. Transport Properties of Nonelectrolyte Liquid Mixtures. XI. Mutual Diffusion Coefficients for Toluene+n-Hexane and Toluene+Acetonitrile at Temperatures from 273 to 348 K and at Pressures up to 25 MPa. *Int. J. Thermophys.* **22**, 679–700 (2001).

[31] Andanson, J. M., Meng, X., Traïkia, M. & Husson, P. Quantification of the impact of water as an impurity on standard physico-chemical properties of ionic liquids. *J. Chem. Thermodyn.* **94**, 169–176 (2016).

[32] Stephan, S., Thol, M., Vrabec, J. & Hasse, H. Thermophysical properties of the Lennard-Jones fluid: Database and data assessment. *J. Chem. Inf. Model.* **59**, 4248–4265 (2019).

[33] Schultz, A. J. & Kofke, D. A. Comprehensive high-precision high-accuracy equation of state and coexistence properties for classical Lennard-Jones crystals and low-temperature fluid phases. *J. Chem. Phys.* **149**, 204508 (2018).

[34] Fertig, D. & Stephan, S. Influence of dispersive long-range interactions on transport and excess properties of simple mixtures. *Mol. Phys.* **121**, e2162993 (2023).

[35] Kolafa, J. & Nezbeda, I. The Lennard-Jones fluid: An accurate analytic and theoretically-based equation of state. *Fluid Phase Equilib.* **100**, 1–34 (1994).

[36] Vignes, A. Diffusion in binary solutions. Variation of diffusion coefficient with composition. *Ind. Eng. Chem. Fund.* **5**, 189–199 (1966).

[37] Krishna, R. & van Baten, J. M. The Darken relation for multicomponent diffusion in liquid mixtures of linear alkanes: An investigation using molecular dynamics (MD) simulations. *Ind. Eng. Chem. Res.* **44**, 6939–6947 (2005).

[38] Liu, X., Vlugt, T. J. & Bardow, A. Predictive Darken equation for Maxwell-Stefan diffusivities in multicomponent mixtures. *Ind. Eng. Chem. Res.* **50**, 10350–10358 (2011).

[39] Fertig, D., Hasse, H. & Stephan, S. Transport properties of binary Lennard-Jones mixtures: Insights from entropy scaling and conformal solution theory. *J. Mol. Liq.* **367**, 120401 (2022).

[40] Rutten, P. W. M. *Diffusion in liquids.* PhD thesis, TU Delft, Delft (1992).

[41] Lötgering-Lin, O., Fischer, M., Hopp, M. & Gross, J. Pure substance and mixture viscosities based on entropy scaling and an analytic equation of state. *Ind. Eng. Chem. Res.* **57**, 4095–4114 (2018).

[42] Li, J., Liu, H. & Hu, Y. A mutual-diffusion-coefficient model based on local composition. *Fluid Phase Equilib.* **187-188**, 193–208 (2001).

[43] He, M., Peng, S., Zhang, Y., Zhang, S. & Liu, X. Mutual diffusion coefficients of isopropanol + n-heptane and isobutanol + n-heptane. *J. Chem. Thermodyn.* **96**, 127–133 (2016).
