# Supplementary material for: Entropy scaling for diffusion coefficients in fluid mixtures
Source: Nat Commun. 2025 Mar 17;16:2611. doi: 10.1038/s41467-025-57780-z (PMC11914492; doi:10.1038/s41467-025-57780-z)
Supplement: Supplementary file 2 — Description of Additional Supplementary Files [file 41467_2025_57780_MOESM2_ESM.pdf]

Description of Additional supplementary file

Supplementary Data 1:

Molecular simulation data the infinite dilution coefficients in the three Lennard-Jones mixtures and the real mixtures acetone + isobutane, benzene + isobutane, and ethanol + chlorine.

Supplementary Software:

The provided code includes an implementation of the new entropy scaling framework in the Julia programming language. Additionally, implementations of both EOS used in this work, namely the PC-SAFT EOS and the Kolafa-Nezbeda EOS, are provided to enable the application of the entropy scaling framework. Two examples demonstrate the usage. The requirements for executing the code, a short documentation, and instructions to run the example are given below.
