# Supplementary figures and images for: Entropy scaling for diffusion coefficients in fluid mixtures
Source: Nat Commun. 2025 Mar 17;16:2611. doi: 10.1038/s41467-025-57780-z (PMC11914492; doi:10.1038/s41467-025-57780-z)

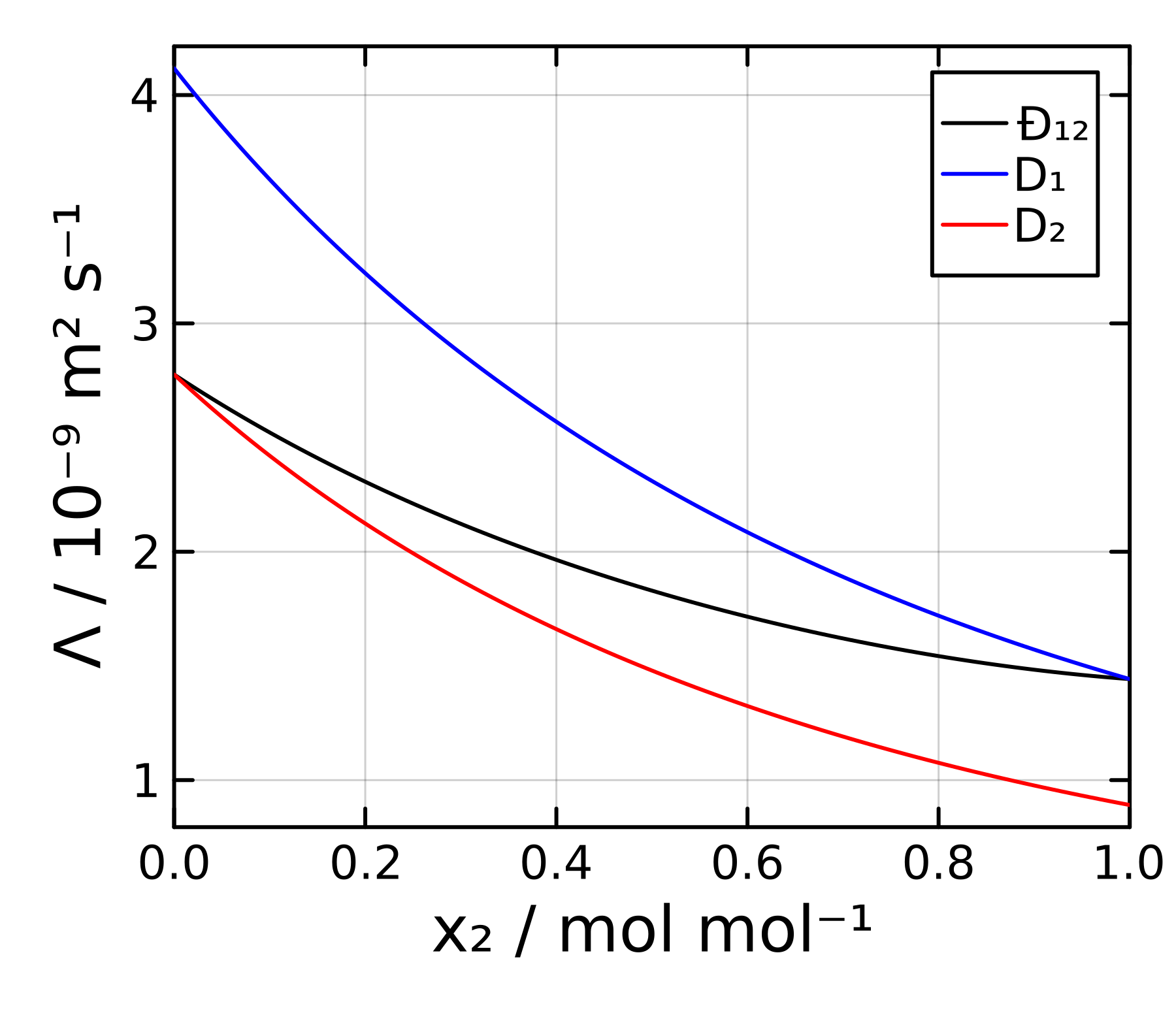

Supplement: Supplementary file 4 — Supplementary Software [file 41467_2025_57780_MOESM4_ESM.zip › example_hexane+dodecane.png]

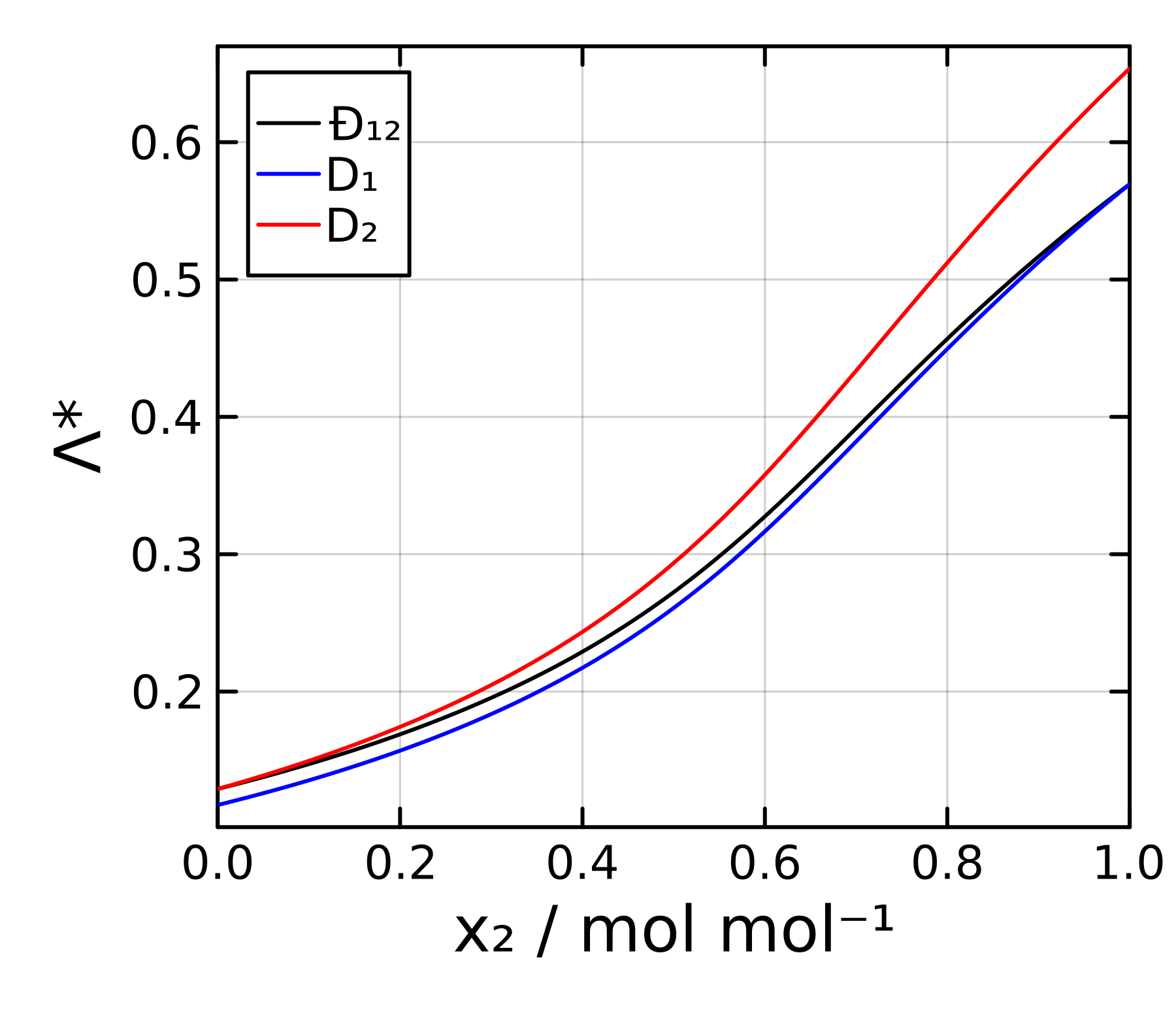

Supplement: Supplementary file 4 — Supplementary Software [file 41467_2025_57780_MOESM4_ESM.zip › example_LJ.png]
